# Dapagliflozin in lupus nephritis: renal and hematologic outcomes from a randomized controlled trial

**DOI:** 10.1093/ckj/sfag231

**Published:** 2026-07-10

**Authors:** Nourelsabah Mohamed, Karem N Zayed, Muhammed Ahmed Elhadedy, Mohamed Hosney Badawi, Wael I Mortada, Kareem A Nabieh, Mohamed A Sobh, Ayman F Refaie

**Affiliations:** Dialysis and Transplantation Unit, Urology and Nephrology Center, Mansoura University, Mansoura, Egypt; Mansoura Nephrology and Dialysis Unit (MNDU), Faculty of Medicine, Mansoura University, Mansoura, Egypt; Dialysis and Transplantation Unit, Urology and Nephrology Center, Mansoura University, Mansoura, Egypt; Dialysis and Transplantation Unit, Urology and Nephrology Center, Mansoura University, Mansoura, Egypt; Clinical Chemistry Laboratory, Urology and Nephrology Center, Mansoura University, Mansoura, Egypt; Clinical Chemistry Laboratory, Urology and Nephrology Center, Mansoura University, Mansoura, Egypt; Dialysis and Transplantation Unit, Urology and Nephrology Center, Mansoura University, Mansoura, Egypt; Dialysis and Transplantation Unit, Urology and Nephrology Center, Mansoura University, Mansoura, Egypt

**Keywords:** dapagliflozin, lupus nephritis, proteinuria, sodium–glucose cotransporter-2 inhibitors

## Abstract

**Background:**

Lupus nephritis (LN) remains a major cause of chronic kidney disease (CKD) and end-stage renal disease despite advances in immunosuppressive therapy. Sodium–glucose cotransporter-2 (SGLT2) inhibitors confer renal protection in diabetic and nondiabetic CKD and have been associated with increases in hemoglobin levels, but their renal and hematologic effects in immune-mediated glomerulopathies such as LN are not well defined.

**Objective:**

To evaluate the renal and hematologic effects, efficacy, and safety of dapagliflozin as adjunctive therapy in patients with LN.

**Methods:**

In this randomized, double-blind, placebo-controlled trial, 79 adult patients with biopsy-proven LN and estimated glomerular filtration rate (eGFR) >30 ml/min/1.73 m^2^ were randomized to receive dapagliflozin 10 mg/day (*n* = 38) or placebo (*n* = 41) for 12 months in addition to standard immunosuppressive therapy. The primary renal endpoint was percentage change in 24-h urinary protein excretion. Secondary renal outcomes included change in eGFR. Hematologic parameters assessed at baseline and 12 months included hemoglobin, erythropoietin, hepcidin, ferritin, and transferrin saturation. The primary analysis used analysis of covariance (ANCOVA) with adjustment for baseline values and clinically relevant covariates including age and background immunosuppressive therapy. Sensitivity analyses using log-transformed proteinuria were also performed. Additional analyses evaluated adjusted 12-month eGFR and eGFR slope.

**Results:**

At 12 months, dapagliflozin was associated with a numerical reduction in proteinuria compared with placebo; however, this difference did not reach statistical significance. Adjusted analyses using ANCOVA confirmed the absence of a significant treatment effect. No significant differences were observed in eGFR, eGFR slope, or hematologic parameters.

**Conclusion:**

In this randomized controlled trial, dapagliflozin was associated with a numerical reduction in proteinuria that did not reach statistical significance after adjustment for baseline characteristics and background immunosuppressive therapy. No significant effect on kidney function, eGFR slope, or hematologic parameters was observed. These findings suggest a possible signal that requires confirmation in larger, adequately powered, longer-duration studies.

Clinical trials registration number: NCT05748925 (ClinicalTrials.gov). Registered 28 February 2023–Retrospectively registered (https://register.clinicaltrials.gov/prs/beta/studies/S000CWI200000138/recordSummary).

KEY LEARNING POINTS **What was known:**

- Large randomized trials established that SGLT2 inhibitors reduce proteinuria and slow kidney disease progression in diabetic and nondiabetic CKD, but patients with active immune-mediated glomerulonephritis, including LN, were systematically excluded, leaving their renal efficacy and safety in this population uncertain.
- LN remains a leading cause of CKD and end-stage renal disease despite modern immunosuppressive therapy, and persistent proteinuria is a major predictor of poor long-term renal outcomes, highlighting the need for effective nonimmunosuppressive adjunctive therapies.
- Emerging evidence suggests SGLT2 inhibitors increase hemoglobin and modulate iron metabolism through erythropoietin and hepcidin pathways in CKD and cardiovascular populations, but their hematologic effects in patients with systemic lupus erythematosus and LN had not been systematically evaluated.

**This study adds:**

- In patients with biopsy-proven LN receiving standard immunosuppressive therapy, adjunctive dapagliflozin over 12 months produced a clinically meaningful reduction in proteinuria compared with placebo, without evidence of short-term deterioration in kidney function or excess adverse events.
- The antiproteinuric effect of dapagliflozin appeared more pronounced among patients with higher baseline proteinuria and moderate renal impairment, suggesting that disease severity may influence responsiveness to SGLT2 inhibition in immune-mediated glomerular disease.
- Although modest numerical increases in hemoglobin and reductions in hepcidin were observed, hematologic benefits did not reach statistical significance, indicating that larger, longer-term studies are required to define the erythropoietic and iron-modulating effects of SGLT2 inhibitors in LN.

**Potential impact:**

- These findings support consideration of SGLT2 inhibitors as a nonimmunosuppressive adjunct to standard therapy in selected patients with LN and persistent proteinuria, potentially expanding treatment options for clinicians seeking additional renoprotective strategies without increasing immunosuppressive burden.
- The demonstrated short-term safety profile of dapagliflozin in an immune-mediated glomerular population may inform clinical guidelines and institutional policies regarding cautious off-label use and monitoring frameworks for SGLT2 inhibitors in LN care pathways.
- Identification of patient subgroups with greater antiproteinuric response may guide personalized treatment algorithms and future policy recommendations, emphasizing risk stratification by baseline proteinuria and kidney function when considering SGLT2 inhibitor therapy in routine practice.

## INTRODUCTION

Systemic lupus erythematosus (SLE) is a chronic autoimmune disease characterized by widespread inflammation and multisystem involvement. The kidneys and hematologic system are among the most frequently affected organs, contributing substantially to morbidity and mortality. Lupus nephritis (LN), a severe renal manifestation of SLE, typically develops within the first 3–5 years of disease onset and remains a leading cause of chronic kidney disease (CKD) and progression to end-stage kidney disease (ESKD) despite advances in immunosuppressive therapy and supportive care [1].

Current treatment strategies for LN focus on immune suppression using corticosteroids and cytotoxic or biologic agents, combined with antiproteinuric and renoprotective measures aimed at preserving renal function [2]. Nevertheless, long-term renal outcomes remain suboptimal, and a significant proportion of patients experience persistent proteinuria or incomplete remission, highlighting the need for adjunctive therapies that provide additional kidney protection.

Sodium–glucose cotransporter-2 (SGLT2) inhibitors, originally developed as glucose-lowering agents for type 2 diabetes mellitus, have demonstrated robust renoprotective effects across a broad spectrum of CKD populations, including nondiabetic patients [3]. These benefits are mediated through multiple mechanisms, including natriuresis, restoration of tubuloglomerular feedback, reduction of intraglomerular pressure, and attenuation of glomerular hyperfiltration. Large, randomized trials have consistently shown reductions in proteinuria and slower decline in estimated glomerular filtration rate (eGFR), generating interest in extending the use of SGLT2 inhibitors to immune-mediated glomerular diseases [4].

However, evidence supporting the use of SGLT2 inhibitors in immune-mediated glomerulonephritis, including LN, remains limited. While preclinical studies and small trials suggest potential benefit, robust randomized controlled data in this population are scarce [5]. Consequently, the renal efficacy and safety of SGLT2 inhibitors in LN remain incompletely defined.

In parallel with renal involvement, hematologic abnormalities are highly prevalent in SLE and may affect all three blood cell lineages, manifesting as anemia, leukopenia, thrombocytopenia, or pancytopenia [6]. Among these, anemia is the most common and clinically significant, frequently resulting from chronic inflammation, iron dysregulation, renal impairment, or medication-related effects. Anemia of chronic disease is the predominant subtype, accounting for nearly one-third of cases in SLE and is largely driven by elevated hepcidin levels induced by inflammatory cytokines such as interleukin-6. Hepcidin inhibits intestinal iron absorption and macrophage iron release, leading to functional iron deficiency and impaired erythropoiesis [7]. Iron deficiency anemia may also occur due to gastrointestinal blood loss or menorrhagia, particularly in patients receiving long-term nonsteroidal anti-inflammatory drugs or corticosteroids. Interpretation of iron indices in SLE is further complicated by ferritin’s role as an acute-phase reactant, which may mask true iron deficiency. Importantly, anemia is not merely a marker of disease activity but an independent contributor to adverse outcomes in LN, CKD, and other chronic inflammatory conditions [8].

Emerging evidence suggests that SGLT2 inhibitors exert hematologic effects beyond plasma volume contraction. A recent meta-analysis demonstrated significant increases in hemoglobin and hematocrit levels with SGLT2 inhibitor therapy compared with placebo [9]. While initial hypotheses attributed this effect to hemoconcentration resulting from osmotic diuresis [10], accumulating data support additional biological mechanisms, including enhanced erythropoietin (EPO) production and improved iron utilization [11].

The EMPA-HEART trial demonstrated early increases in EPO and hematocrit within 1 month of empagliflozin initiation, suggesting stimulation of erythropoiesis rather than simple volume contraction [12]. EPO is produced by renal interstitial fibroblasts in response to tissue hypoxia via hypoxia-inducible factor (HIF) signaling pathways [13], and SGLT2 inhibitors may influence HIF metabolism directly, as suggested by experimental models of diabetic kidney disease [14]. Furthermore, dapagliflozin has been reported to suppress hepcidin levels, potentially through EPO-mediated induction of erythroferrone, which downregulates hepatic hepcidin transcription [15]. This pathway may enhance iron mobilization and availability, reflected by reductions in ferritin and increases in transferrin saturation (TSAT) and transferrin receptor expression [16].

Given the coexistence of renal and hematologic involvement in LN, and the emerging pleiotropic effects of SGLT2 inhibitors, this study was designed to evaluate the efficacy and safety of dapagliflozin as adjunctive therapy in patients with biopsy-confirmed LN. Specifically, we aimed to assess its impact on renal outcomes—particularly proteinuria and kidney function—as well as hematologic parameters, including hemoglobin, EPO, iron indices, and hepcidin levels, over a 12-month period.

## MATERIALS AND METHODS

This prospective, randomized, double-blind, placebo-controlled, parallel-group trial was conducted at the Nephrology Clinic of the Urology and Nephrology Center, Mansoura University, Egypt, between October 2022 and November 2023. The study aimed to evaluate the efficacy and safety of dapagliflozin in adult patients with biopsy-confirmed LN, assessing both renal and hematologic outcomes over 12 months in addition to standard immunosuppressive therapy.

### Study population and recruitment

Adult patients (≥18 years) with SLE fulfilling the 2019 European League Against Rheumatism/American College of Rheumatology classification criteria and biopsy-proven LN were eligible. Participants were required to have an eGFR >30 ml/min/1.73 m^2^ at baseline. Patients with or without diabetes mellitus were eligible. Key exclusion criteria included eGFR <30 ml/min/1.73 m^2^, pregnancy or breastfeeding, significant comorbidities affecting renal or hematologic outcomes (e.g. chronic liver disease, malignancy, severe respiratory, or gastrointestinal illness), urinary tract obstruction, concurrent use of both angiotensin-converting enzyme (ACE) inhibitors and angiotensin receptor blockers (ARBs), and frequent hypotension (systolic blood pressure <100 mmHg). All participants provided written informed consent prior to enrollment.

### Randomization and masking

Participants were randomized in a 1:1 ratio to receive dapagliflozin or matching placebo, stratified by age (≤50 vs >50 years) and gender, using computer-generated random sequences in SPSS v22. Allocation concealment was maintained via a secure web-based system, accessible only to the designated pharmacist responsible for dispensing study medications. Both participants and study personnel were blinded to group assignment throughout the study, with identical packaging, labeling, appearance, taste, and smell of the active drug and placebo.

### Interventions

**Dapagliflozin group:** In this, 38 participants received 10 mg of dapagliflozin once daily.

**Placebo group:** In this, 41 participants received matching placebo.

All randomized participants were included in the final analysis according to the intention-to-treat principle. Five participants discontinued follow-up (four in the dapagliflozin group and one in the placebo group), all due to refusal to continue.

### Follow-up and data collection

Participants were evaluated at baseline and at 1, 3, 6, 9, and 12 months. Clinical assessments, medication adherence (via pill count), and adverse events (AEs) were documented at each visit. Baseline data included demographics, comorbidities, immunosuppressive regimens, antihypertensive therapy, adjunctive medications (ACE inhibitors, ARBs, diuretics, and statins), renal biopsy findings (class, activity, and chronicity scores), and disease duration.

### Renal assessments

The primary renal outcome was the percentage change in 24-h urinary protein excretion from baseline to 12 months. Secondary renal outcomes included change in eGFR, incidence of AEs [including acute kidney injury (AKI) and urinary or genital infections], and progression to kidney failure (ESKD). Serum creatinine and urinary protein were measured using Abbott ARCHITECT c4000®, and eGFR was calculated using the Chronic Kidney Disease Epidemiology Collaboration (CKD-EPI) formula.

### Hematologic assessments

Hematologic parameters were assessed at baseline and at 3-, 6-, 9-, and 12-month visits. These included complete blood count (CBC) measures—hemoglobin, mean corpuscular volume (MCV), mean corpuscular hemoglobin (MCH), and platelet count—as well as iron profile (serum ferritin and TSAT). Serum EPO and hepcidin levels were measured at baseline and at 12 months to evaluate changes in erythropoietic activity. CBC analyses were performed using the Sysmex XN-1000®, iron profile using Roche COBAS 6000®, and serum EPO and hepcidin using ELISA kits (Chongqing Bioses®, China).

### Sample size calculation

A total of 84 participants were enrolled to detect a clinically relevant difference in primary hematologic and renal outcomes, accounting for potential attrition. A sample size of 38 participants per group (total *n* = 76) provided 90% power to detect a standardized effect size of 0.100 for hematologic parameters at α = 0.05.

### Statistical analysis

Continuous variables were assessed for normality using the Shapiro–Wilk test and presented as mean ± standard deviation (SD) or median [interquartile range (IQR)] as appropriate. Categorical variables were summarized as counts and percentages. Between-group comparisons were performed using Student’s t-test or Mann–Whitney U test for continuous variables and chi-square or Fisher’s exact test for categorical variables. The primary analysis was performed using analysis of covariance (ANCOVA), with 12-month proteinuria as the dependent variable and treatment group as the main factor, adjusting for baseline proteinuria, baseline eGFR, age, and background immunosuppressive therapy. Sensitivity analyses were conducted using log-transformed proteinuria. Additional ANCOVA models were performed for 12-month eGFR and eGFR slope. Multivariate linear regression was used to assess predictors of hematologic outcomes. A two-sided *P* < .05 was considered statistically significant. Analyses were performed using SPSS v22 and RStudio.

### Ethical considerations and trial registration

The study adhered to the Declaration of Helsinki and Good Clinical Practice guidelines. Ethical approval was obtained from the Institutional Review Board of the Faculty of Medicine, Mansoura University (Approval No: MD.21.10.550-9/11/2021). The study was registered at ClinicalTrials.gov (NCT05748925). The trial followed the Consolidated Standards of Reporting Trials (CONSORT) 2010 guidelines.

## RESULTS

### Recruitment and follow-up

Participants were enrolled between 15 September 2022 and 30 November 2023. Follow-up continued for 12 months after randomization. The trial was completed as planned without being terminated prematurely.

**Figure 1:**
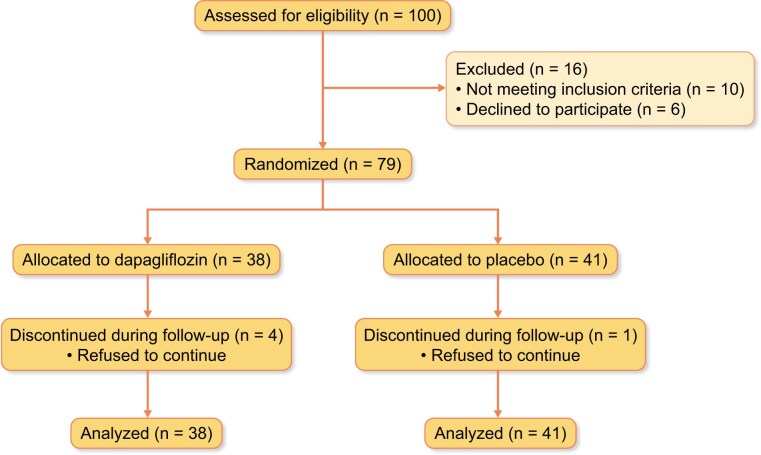
CONSORT flow diagram of patient enrollment, randomization, follow-up, and analysis. Of 100 patients assessed for eligibility, 16 were excluded. Total 79 patients were randomized (38 to dapagliflozin and 41 to placebo). During follow-up, five patients discontinued the study (four in the dapagliflozin group and one in the placebo group), all due to refusal to continue. All random patients were included in the final analysis.

A total of 100 patients were assessed for eligibility, of whom 16 were excluded. Total 79 patients were randomized (38 to dapagliflozin and 41 to placebo). The flow of participants through the study is shown in Fig. 1. Baseline demographic and clinical characteristics were generally well-balanced between the two groups (Table 1), supporting the internal validity of the trial design.

**Table 1:** Demographic and clinical characteristics of the participants at baseline.

	Drug *N* = 38	Placebo *N* = 41	*P*-value
Age	38 ± 10	37 ± 10	.51
Sex			.40
Female	34 (89%)	34 (83%)	
Male	4 (11%)	7 (17%)	
Body mass index (kg/m^2^)	33 ± 6	31 ± 11	.18
Estimated GFR	96 (58–113)	93 (58–116)	.86
Estimated GFR distribution			.90
≥60 ml/min/1.73 m^2^	28 (74%)	29 (71%)	
45 to <60 ml/min/1.73 m^2^	4 (11%)	7 (17%)	
30 to <45 ml/min/1.73 m^2^	4 (11%)	3 (7.3%)	
<30 ml/min/1.73 m^2^	2 (5.3%)	2 (4.9%)	
Hemoglobin—g/dl	11.66 ± 1.70	12.00 ± 2.00	.42
Serum potassium—mEq/l	4.01 ± 0.41	3.96 ± 0.48	.64
Urinary protein in 24 h/day	0.60 (0.40–1.00)	0.40 (0.20–1.60)	.31
Urinary protein >1 gm/day	10 (26%)	13 (32%)	.60
Diabetes mellitus	11 (29%)	7 (17%)	.21
Hypertension	38 (100%)	37 (90%)	.12
Antiphospholipid syndrome	3 (7.9%)	6 (15%)	.48
Biopsy-proven LN class			.067
1	1 (2.6%)	1 (2.4%)	
2	7 (18%)	2 (4.9%)	
3	8 (21%)	11 (27%)	
4	16 (42%)	26 (63%)	
5	5 (13%)	1 (2.4%)	
6	1 (2.6%)	0 (0%)	
Immunosuppressive regimen			.20
st + aza	14 (37%)	7 (17%)	
st + cni	4 (11%)	5 (12%)	
st + cni + mmf	1 (2.6%)	2 (4.9%)	
st + mmf	18 (47%)	27 (66%)	
Steroid	1 (2.6%)	0 (0%)	
Cyclophosphamide	5 (13%)	7 (17%)	.63
ACE inhibitor	27 (71%)	26 (63%)	.47
Diuretics	16 (42%)	14 (34%)	.47
Statins	24 (63%)	30 (73%)	.34
Disease duration—years	7.0 (4.0 –12.0)	6.0 (3.0 –11.0)	.37

Values are mean ± SD, median (Q1–Q3), or *n* (%). *P*-values from the Welch two–sample t test, the Wilcoxon rank–sum test, the Pearson χ² test, or Fisher’s exact test, as appropriate.

The mean (± SD) age was 38 ± 10 years in the dapagliflozin group and 37 ± 10 years in the placebo group (*P* = .51). Most participants were female (89% vs 83%, *P* = .40). The median (Q1, Q3) eGFR was 96 (58, 113) ml/min/1.73 m^2^ in the dapagliflozin group and 93 (58, 116) ml/min/1.73 m^2^ in the placebo group (*P* = .86). The distribution of patients across eGFR categories (≥60, 45 to <60, 30 to <45, and <30 ml/min/1.73 m^2^) was comparable between groups (*P* = .90).

The median (Q1, Q3) 24-h urinary protein excretion was 0.60 (0.40, 1.00) g/day in the dapagliflozin group and 0.40 (0.20, 1.60) g/day in the placebo group (*P* = .31). The proportions of patients with urinary protein levels >1 g/day were 26% and 32%, respectively (*P* = .60).

Hypertension was present in all patients in the dapagliflozin group (100%) and in 90% of those in the placebo group (*P* = .12), while diabetes mellitus was observed in 29% and 17%, respectively (*P* = .21). Antiphospholipid syndrome was present in 7.9% of the dapagliflozin group and 15% of the placebo group (*P* = .48).

Based on renal biopsy findings, the distribution of LN classes (I–VI) was not significantly different between groups (*P* = .067). The use of various immunosuppressive regimens was also comparable (*P* = .20), including combinations such as steroid plus mycophenolate mofetil (47% vs 66%) and steroid plus azathioprine (37% vs 17%). Use of specific medications such as cyclophosphamide (13% vs 17%, *P* = .63), ACE inhibitors (71% vs 63%, *P* = .47), diuretics (42% vs 34%, *P* = .47), and statins (63% vs 73%, *P* = .34) did not differ significantly. The median (Q1, Q3) disease duration was 7.0 (4.0, 12.0) years in the dapagliflozin group and 6.0 (3.0, 11.0) years in the placebo group (*P* = .37).

AEs were actively monitored at each study visit and documented using standardized definitions based on Common Terminology Criteria for Adverse Events version 5.0. Investigators assessed the severity and attribution of each event. AEs were recorded regardless of suspected causality. AKI was defined as an increase in serum creatinine of ≥0.3 mg/dl within 48 h or ≥1.5-fold from baseline within 7 days, consistent with Kidney Disease: Improving Global Outcomes 2012 criteria.

Table 2 summarizes all reported AEs by treatment group. The overall incidence of AEs was comparable between the dapagliflozin and placebo groups (31.6% vs 26.8%). No serious AEs were reported in either group. The most observed events included AKI (18.4% vs 7.3%) and urinary tract infections (18.4% vs 14.6%) in the dapagliflozin and placebo groups, respectively. Genital infections were infrequent (5.3% vs 2.4%), and progression to kidney failure (ESKD) occurred in one patient in each group. No statistically significant differences in AE rates were observed between groups. No new safety signals were identified during the study period.

**Table 2:** AEs by treatment group.

AE	Dapagliflozin (*n* = 38)	Placebo (*n* = 41)	*P*-value
Any AE	12 (31.6%)	11 (26.8%)	–
Serious AEs	0 (0%)	0 (0%)	–
AKI	7 (18.4%)	3 (7.3%)	–
Urinary tract infection	7 (18.4%)	6 (14.6%)	–
Genital infection	2 (5.3%)	1 (2.4%)	–
Progression to ESRD	1 (2.6%)	1 (2.4%)	–

Values are presented as number (%). Serious AEs were defined according to standard clinical criteria. No statistically significant differences were observed between groups.

### Change in 24-h urine protein over 12 months

**Figure 2:**
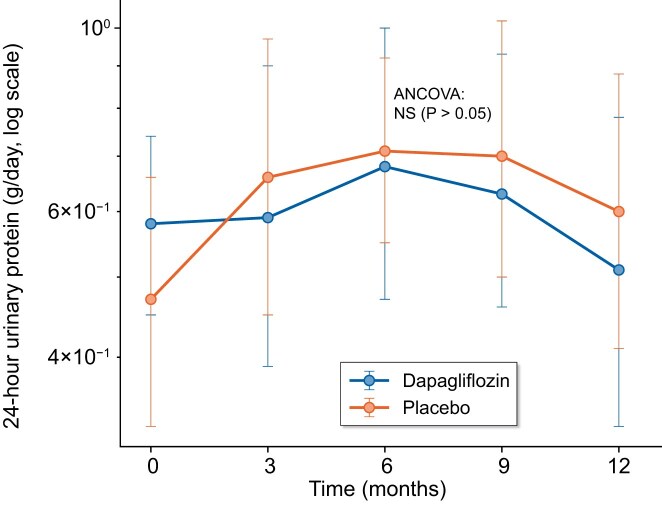
24–h urine protein excretion over 12 months. Change in proteinuria over time in the dapagliflozin and placebo groups. Proteinuria values are presented as geometric means with 95% confidence intervals on a logarithmic scale. No statistically significant difference was observed between groups at 12 months. Adjusted analysis using ANCOVA did not demonstrate a significant treatment effect.

Figure 2 shows the geometric mean of 24-h urine protein excretion over 12 months. At baseline, proteinuria was 0.58 g/24 h in the dapagliflozin group and 0.48 g/24 h in the placebo group. In the placebo group, proteinuria increased to a peak of 0.72 g/24 h at Month 6 and declined slightly to 0.61 g/24 h by Month 12. In contrast, proteinuria in the dapagliflozin group rose modestly to 0.68 g/24 h at Month 6, then decreased steadily to 0.50 g/24 h at Month 12.

At 12 months, dapagliflozin was associated with a numerical reduction in proteinuria compared with placebo. However, the between-group difference did not reach statistical significance. In adjusted analyses using ANCOVA, accounting for baseline proteinuria, baseline eGFR, age, and background immunosuppressive therapy, no statistically significant difference in 12-month proteinuria was observed between groups.

Sensitivity analyses using log-transformed proteinuria yielded consistent findings, with baseline proteinuria remaining the strongest predictor of 12-month proteinuria (*P* < .001).

### Change in estimated glomerular filtration rate over 12 months

**Figure 3:**
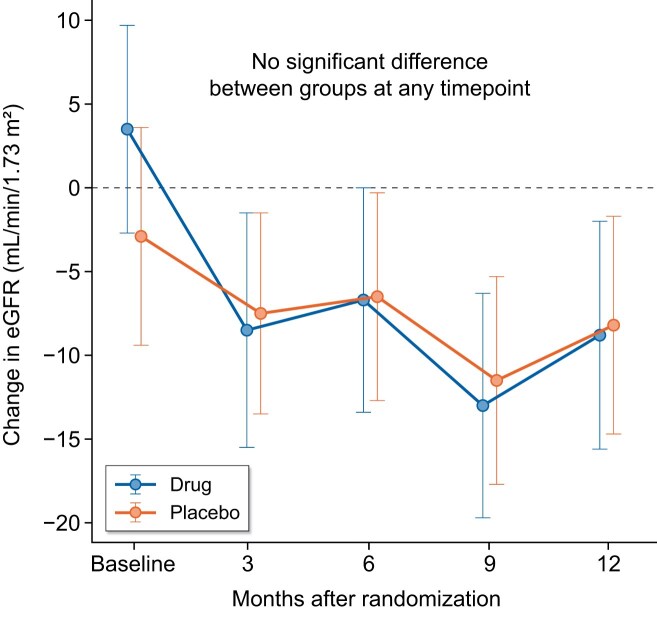
Change in eGFR over 12 months. Least–squares mean change ± 95 % confidence intervals in eGFR for participants receiving dapagliflozin (blue) or placebo (orange). Negative values indicate decline from the baseline. The dashed horizontal line denotes no change. A mixed-effects repeated–measures analysis showed no significant between–group difference during any scheduled visit. Vertical bars represent confidence intervals; points are plotted at baseline, 3, 6, 9, and 12 months after randomization.

Figure 3 displays the mean change in eGFR from baseline over a 12-month follow-up period in patients randomized to receive either dapagliflozin or placebo. Both groups experienced a decline in eGFR with no significant differences at any time point. By Month 12, the mean reduction in eGFR was 8.74 ml/min/1.73 m^2^ in the dapagliflozin group and 8.32 ml/min/1.73 m^2^ in the placebo group.

The pattern of eGFR change was marked by fluctuation in both groups. Initial declines were noted in 3 months, followed by partial recovery at 6 months, a further drop at 9 months, and modest improvement by Month 12. Confidence intervals overlapped throughout, and statistical analysis confirmed the absence of a significant treatment effect over time. No statistically significant difference in eGFR or eGFR slope was observed between groups in adjusted or unadjusted analyses. These findings indicate no observable short-term difference in kidney function between groups as measured by eGFR.

**Figure 4:**
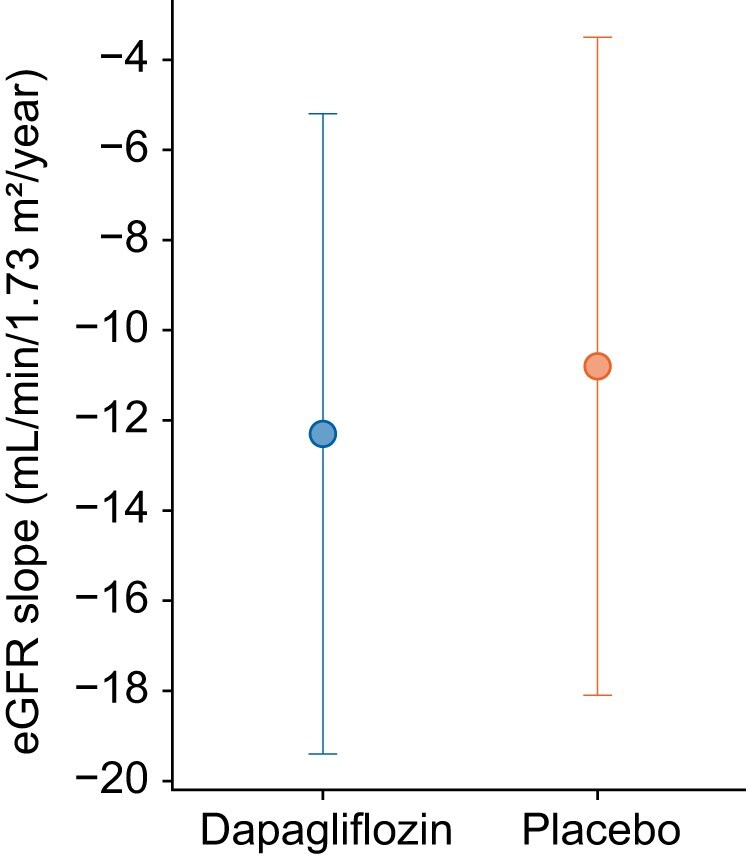
Annualized eGFR slope over 12 months in the dapagliflozin and placebo groups. Annualized eGFR slope over 12 months in the dapagliflozin and placebo groups. Values are presented as mean with 95% confidence intervals. No statistically significant difference was observed between groups.

In adjusted analyses using ANCOVA, no statistically significant difference in 12-month eGFR was observed between treatment groups. Baseline eGFR was the strongest predictor of follow-up kidney function (*P* < .001). The rate of change in eGFR over time was also evaluated. The annualized eGFR slope over 12 months was comparable between groups, with overlapping 95% confidence intervals. These findings were consistent with adjusted analyses, which showed no statistically significant difference between treatment groups (Fig. 4).

### Subgroup analysis based on baseline kidney function and proteinuria

**Figure 5:**
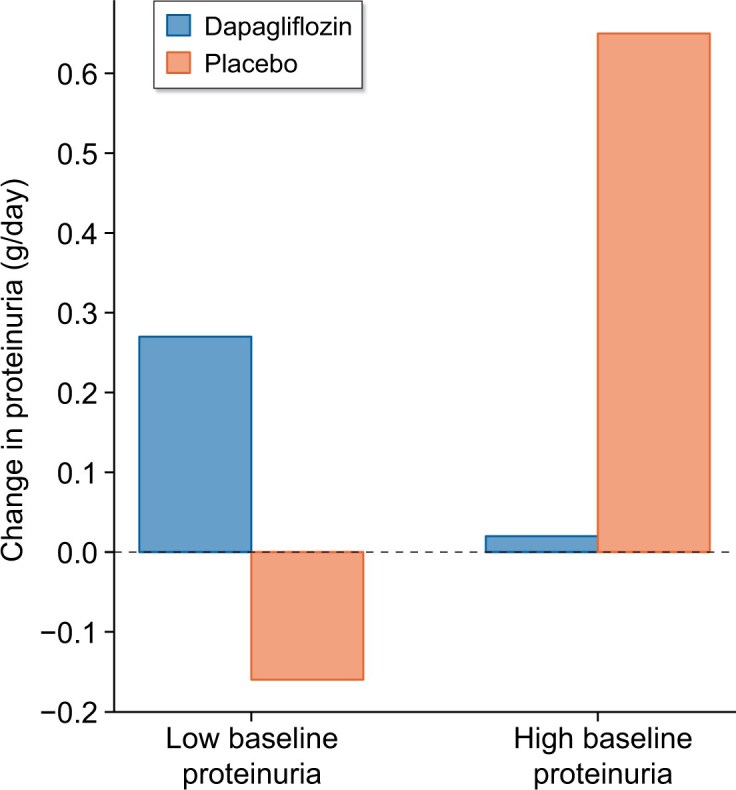
Exploratory subgroup analysis of proteinuria response. Exploratory subgroup analysis of proteinuria response according to baseline kidney function and proteinuria. Findings are exploratory and should be interpreted with caution. No statistically significant interaction was observed.

Figure 5 presents subgroup analyses comparing the effect of dapagliflozin versus placebo on the primary outcome (percentage change in 24-h urinary protein excretion) in patients with LN, stratified by baseline eGFR and proteinuria. These post hoc exploratory analyses were not prespecified and should be interpreted as hypothesis-generating only.

Exploratory subgroup analyses suggested a possible trend toward greater antiproteinuric response in patients with higher baseline proteinuria or moderate renal impairment; however, no statistically significant interaction was observed, and these findings should be interpreted with caution.

### Hematologic parameters over time

Table 3 presents the effect of the intervention on hematological parameters (hemoglobin, MCV, and MCH) in both the control and dapagliflozin groups across different time points (baseline, 3, 6, 9, and 12 months). No statistically significant differences between group were observed in hemoglobin, MCV, or MCH at any time point over the 12-month follow-up period.

**Table 3:** Hematologic parameters at baseline and 12 months.

Variable	Dapagliflozin (*n* = 38)	Placebo (*n* = 41)	*P*-value
Hemoglobin baseline (g/dl)	11.66 ± 0.28	12.00 ± 0.31	NS
Hemoglobin 12 months (g/dl)	12.21 ± 0.34	12.23 ± 0.31	NS
MCV baseline (fl)	84.11 ± 1.13	82.84 ± 1.10	NS
MCV 12 months (fl)	84.32 ± 1.29	81.50 ± 2.13	NS
MCH baseline (pg)	26.34 ± 0.48	25.43 ± 0.46	NS
MCH 12 months (pg)	27.12 ± 0.55	26.97 ± 0.50	NS

Values are presented as mean ± standard error. No statistically significant between-group differences were observed. NS: not statistically significant.

### Anemia prevalence and response

**Figure 6:**
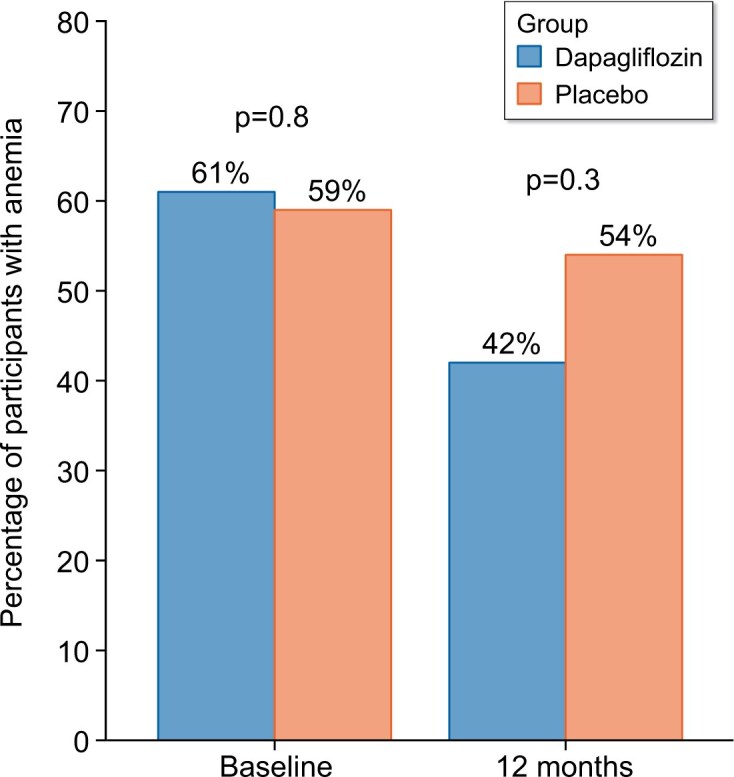
Percentage of anemic participants before and after intervention in both groups. Axis labels: x-axis indicates timepoints (pre-/postintervention); y-axis shows percentage values. Prevalence of anemia at baseline and after 12 months in the dapagliflozin and placebo groups. Bars represent the percentage of participants meeting criteria for anemia at baseline and at the 12-month follow-up. At baseline, anemia prevalence was similar between groups (61% in the dapagliflozin group vs 59% in the placebo group; *P* = .8). After 12 months, anemia prevalence decreased in the dapagliflozin group (42%) and remained higher in the placebo group (54%), with no statistically significant between-group difference.

In terms of anemia incidence, there were no significant differences observed between the drug and placebo groups before and after the intervention. Prior to the intervention, 60.5% of participants in the drug group and 58.5% of participants in the placebo group were classified as anemic. After the intervention, 42.1% of participants in the drug group and 53.7% in the placebo group were anemic. The prevalence of anemia decreased in both groups over time; however, no statistically significant differences were observed between groups (Fig. 6).

### Iron and erythropoietic markers

Table 4 presents the between-group differences in other hematological parameters, including ferritin, TSAT, EPO, hepcidin, and platelet count, at baseline and 12 months. No statistically significant differences between group were observed in ferritin, TSAT, EPO, hepcidin, or platelet count.

**Table 4:** Iron metabolism and erythropoiesis markers.

Variable	Dapagliflozin (*n* = 38)	Placebo (*n* = 41)	*P*-value
Ferritin (ng/ml)			
Baseline, median (IQR)	81 [33, 160]	51 [25, 136]	.114
12 months, median (IQR)	64 [28, 127]	75 [36, 175]	.451
TSAT (%)			
Baseline, median (IQR)	23.5 [17.1, 28.2]	23 [15.3, 34]	.837
12 months, median (IQR)	20 [6, 66]	28 [16, 38]	.114
EPO (IU/l)			
Baseline, mean ± SD	11.4 ± 1.89	11.6 ± 2.1	.496
12 months, mean ± SD	12.5 ± 3.31	12.8 ± 3.5	.692
Hepcidin (ng/ml)			
Baseline, mean ± SD	182 ± 76	198 ± 74	.335
12 months, mean ± SD	116 ± 15	128 ± 63	.293
Platelet count (×10^3^/µl)			
Baseline, mean ± SD	295 ± 79	288 ± 71	.630
12 months, mean ± SD	281 ± 72.6	283 ± 74	.506

Values are presented as mean ± SD or median (IQR) as appropriate. *P*-values represent between-group comparisons. No statistically significant between-group differences were observed.

### Predictors of hemoglobin response

Table 5 presents regression analysis identifying baseline serum hemoglobin and MCV as strong predictors of hemoglobin changes. Baseline hemoglobin was significantly associated with hemoglobin changes in both univariate and multivariate models, with a beta coefficient of 0.91 (*P* < .001) in the univariate model and 0.86 (*P* < .001) in the multivariate model, indicating its association with changes in hemoglobin levels. Similarly, baseline MCV showed a significant association with hemoglobin changes, with a beta coefficient of 0.12 (*P* < .001) in the univariate model and 0.05 (*P* = .014) in the multivariate model, suggesting a potential association with hemoglobin variability (Table 5).

**Table 5:** Multivariable regression analysis for predictors of hemoglobin at 12 months.

Variable	β Coefficient	95% CI	*P*-value
Baseline hemoglobin	0.86	(0.72–1.00)	<.001
MCV	0.05	(0.02–0.09)	.006
Treatment group (dapagliflozin)	−0.19	(−0.68 to 0.31)	.461

Model R^2^ = 0.724, adjusted R^2^ = 0.713.

Treatment assignment was not significantly associated with hematologic outcomes. CI: confidence interval.

Other factors, such as age, baseline TSAT, EPO, serum hepcidin, and eGFR, were not significant predictors of hemoglobin changes in multivariate analysis (*P* > .05), indicating no significant independent association with hemoglobin changes. The intervention (dapagliflozin vs placebo) also showed no significant effect on hemoglobin levels in either model, indicating that the drug did not significantly influence the hematological outcomes. Although baseline TSAT was significant in univariate analysis (β = 0.03, *P* = .033), this effect was not retained after adjusting for other variables (Table 5).

To identify potential specific predictors of hemoglobin levels related to SLE and its presentation in patients, univariate and multivariate regression analyses were performed (Table 6). Multivariable regression analysis identified baseline hemoglobin and MCV as significant predictors of hemoglobin at 12 months. Treatment assignment was not independently associated with hematologic outcomes.

**Table 6:** Multivariable regression analysis for predictors of change in hemoglobin over 12 months.

Variable	β Coefficient	95% CI	*P*-value
Baseline hemoglobin	−0.14	(−0.28 to −0.00)	.045
MCV	0.05	(0.02–0.09)	.006
Treatment group (dapagliflozin)	−0.19	(−0.68 to 0.31)	.461

Model R^2^ = 0.131.

Treatment assignment was not significantly associated with hemoglobin change. CI: confidence interval.

In adjusted analyses using ANCOVA, no statistically significant differences were observed between groups for proteinuria at 12 months, eGFR at 12 months, or annualized eGFR slope (Table 7). These results remained consistent after adjustment for baseline eGFR, baseline proteinuria, and age, supporting the robustness of the primary findings.

**Table 7:** Adjusted analysis of primary and secondary renal outcomes (ANCOVA).

Outcome	Adjusted difference	95% CI	*P*-value
Proteinuria (12 months)	0.10	(−0.47 to 0.68)	.720
eGFR (12 months)	−1.95	(−12.29 to 8.38)	.708
eGFR slope	−1.13	(−10.47 to 8.21)	.810

Adjusted for baseline eGFR, baseline proteinuria, and age (where available). No statistically significant differences were observed between groups. CI: confidence interval.

## DISCUSSION

In this 12-month randomized trial, dapagliflozin was associated with a numerical reduction in proteinuria compared with placebo; however, this difference did not reach statistical significance. Adjusted analyses and sensitivity analyses using log-transformed proteinuria yielded consistent findings. No significant difference was observed in eGFR, 12-month adjusted kidney function, or eGFR slope between groups. These preliminary findings require confirmation in larger, adequately powered studies with longer follow-up.

Large, randomized trials in diabetic and nondiabetic CKD have established the kidney-protective effects of SGLT2 inhibitors. However, patients with active immune-mediated glomerulonephritis, including LN, were largely excluded from these studies. Our trial therefore addresses an important evidence gap by evaluating dapagliflozin specifically in biopsy-proven LN. The CREDENCE trial was the first to show a marked reduction in kidney failure risk in patients with diabetic nephropathy treated with canagliflozin [17]. The DAPA-CKD trial later extended these findings to a broader CKD population, including nondiabetics, demonstrating a ∼39% relative risk reduction in a composite renal outcome with dapagliflozin​ [18]. Similarly, the EMPA-KIDNEY trial showed that empagliflozin lowered the risk of kidney disease progression or cardiovascular death by 28% in a diverse CKD cohort [19]. Notably, patients with active immune-mediated glomerulonephritis, such as LN, were excluded from these landmark trials, leaving a significant gap in the evidence base [20]. Our study contributes to closing this gap by focusing specifically on LN, a condition not previously represented in SGLT2 inhibitors trials.

We observed a numerical reduction in proteinuria in the dapagliflozin group over 12 months; however, the between-group difference did not reach statistical significance. Importantly, adjusted analyses accounting for baseline proteinuria, baseline eGFR, age, and background immunosuppressive therapy did not demonstrate a significant treatment effect, and sensitivity analyses using log-transformed proteinuria yielded consistent findings. These results suggest a possible signal that should be interpreted cautiously.

Previous small studies in LN have reported reductions in proteinuria with SGLT2 inhibitors [21, 22]; however, these findings have been inconsistent and based on limited sample sizes.

Proteinuria remains one of the most critical surrogate markers in kidney disease, including LN, and its reduction is widely associated with improved long-term outcomes [23, 24]. Thus, though modest in scale, the decrease we observed is nonetheless clinically encouraging.

No statistically significant difference in eGFR was observed between groups over 12 months, either in unadjusted analyses or after adjustment for baseline eGFR, baseline proteinuria, age, and background immunosuppressive therapy. Similarly, no significant difference in eGFR slope was detected. These findings may reflect the relatively short duration of follow-up, as changes in kidney function often require longer observation periods to become apparent. In large clinical trials such as DAPA-CKD and EMPA-KIDNEY, differences in kidney function trajectories typically became evident after longer follow-up periods [20, 30].

Pathophysiological differences between LN and diabetic kidney disease may partly explain why findings in LN do not necessarily mirror those observed in diabetic CKD trials. LN is primarily driven by immune complex deposition and inflammatory injury [26], whereas diabetic kidney disease is more strongly influenced by hemodynamic and metabolic factors [27]. SGLT2 inhibitors may therefore address only part of the pathway contributing to kidney damage in LN [28].

Any potential hemodynamic effect of dapagliflozin may have been attenuated by the dominant contribution of ongoing immune-mediated injury and concurrent immunosuppressive treatment.

Follow-up duration is an important consideration when interpreting these findings.

SGLT2 inhibitors may exert effects beyond hemodynamic changes, including potential anti-inflammatory, antifibrotic, and metabolic actions [32]. Experimental studies in LN models have suggested possible protective effects on podocyte structure and glomerular inflammation [22]. However, our study did not demonstrate a significant treatment effect, and such mechanisms remain speculative in this clinical context, particularly in the presence of concurrent immunosuppressive therapy [22, 32].

In nonlupus populations, SGLT2 inhibitors have been consistently associated with increases in hemoglobin and hematocrit, as well as reductions in anemia incidence in patients with diabetes and CKD [33–35]. These observations provided a rationale for evaluating hematologic outcomes in our LN cohort [34, 35].

In our study, no statistically significant between-group differences were observed in hemoglobin levels or anemia prevalence. Although numerical changes were noted over time, these findings should be interpreted cautiously. Similar observations have been reported in small studies of LN; however, the available evidence remains limited and inconsistent [36].

Mechanistic studies suggest that SGLT2 inhibitors may influence erythropoiesis through effects on EPO signaling, iron handling, and tissue oxygenation pathways [37, 38]. In our study, however, no statistically significant between-group differences were observed in EPO levels. These findings indicate that such mechanisms were not demonstrable in this cohort and should be interpreted cautiously [37, 38].

Although changes in hepcidin levels were observed over time, no statistically significant between-group differences were identified. These findings do not support a clear effect of dapagliflozin on iron regulation in this cohort. Previous studies in CKD and diabetic populations have reported reductions in hepcidin with SGLT2 inhibitors [39]; however, such effects were not demonstrable in our study [39].

Multivariable regression analysis identified baseline hemoglobin and MCV as significant predictors of hemoglobin at 12 months, while treatment allocation, LN class, disease activity, and complement status were not independently associated with hematologic outcomes. These findings indicate that baseline hematologic parameters were associated with subsequent hemoglobin levels. Although TSAT <20% was associated with lower hemoglobin in univariate analysis, this association was not retained in multivariable models [40].

Whether SGLT2 inhibitors can influence renal repair, histologic remission, or fibrosis progression in LN remains uncertain and will require future studies incorporating mechanistic endpoints such as urinary biomarkers, gene expression profiling, or protocol biopsies.

This study has several limitations. First, the sample size was modest, limiting statistical power to detect small-to-moderate treatment effects, particularly for hematologic outcomes, subgroup analyses, and interaction testing. Second, the 12-month follow-up was likely insufficient to evaluate longer-term renal outcomes such as sustained eGFR preservation or progression to kidney failure (ESKD). Third, proteinuria is inherently variable and influenced by disease activity, hemodynamic factors, and background immunosuppressive therapy; although sensitivity analyses using log-transformed proteinuria were performed, the findings should be interpreted cautiously. Fourth, lupus disease activity measures such as the Systemic Lupus Erythematosus Disease Activity Index (SLEDAI) and the British Isles Lupus Assessment Group (BILAG) ) were not systematically collected at all time points and therefore could not be formally adjusted for in the primary analyses; variations in disease activity over the follow-up period may have influenced proteinuria and eGFR measurements. Fifth, exploratory subgroup findings should be considered hypothesis-generating only. Finally, as a single-center study conducted in a tertiary academic setting, generalizability to broader populations may be limited.

Despite these limitations, our study provides randomized data in a population largely excluded from major SGLT2 inhibitor trials. The safety profile of dapagliflozin was acceptable, with no unexpected AEs, although the study was not powered to detect rare complications.

As a single-center study, generalizability may be limited, particularly to patients with advanced renal impairment, active systemic disease flares, or different immunosuppressive regimens. Further multicenter studies in more diverse populations are needed to validate these findings.

## CONCLUSION

In this randomized, double-blind, placebo-controlled trial, dapagliflozin used as adjunctive therapy for 12 months in patients with LN was associated with a numerical reduction in proteinuria that did not reach statistical significance after adjustment. No significant effect on kidney function, eGFR slope, or hematologic parameters was observed. These findings suggest a possible signal that requires confirmation in larger, adequately powered studies with longer follow-up. Further multicenter trials are needed to clarify the role of SGLT2 inhibitors in LN.

## Data Availability

The datasets generated and analyzed during the current study are available from the corresponding author on reasonable request.

## References

[1] Siegel CH, Sammaritano LR. Systemic lupus erythematosus: a review. JAMA. 2024;331:1480–91. 10.1001/jama.2024.2315 38587826

[2] Papachristodoulou E, Kyttaris VC. New and emerging therapies for systemic lupus erythematosus. Clin Immunol. 2024;263:110200. 10.1016/j.clim.2024.110200 38582250

[3] McLean P, Bennett J, “Trey” Woods E et al. SGLT2 inhibitors across various patient populations in the era of precision medicine: the multidisciplinary team approach. npj Metab Health Dis. 2025;3:29. 10.1038/s44324-025-00068-z

[4] Koh ES, Kim GH, Chung S. Intrarenal mechanisms of sodium-glucose cotransporter-2 inhibitors on tubuloglomerular feedback and natriuresis. Endocrinol Metab. 2023;38:359–72. 10.3803/EnM.2023.1764 PMC10475968 37482684

[5] Karacabeyli D, Lacaille D. SGLT-2 inhibitors for the prevention of autoimmune rheumatic diseases. BMJ. 2025;; 391:r2121. 10.1136/bmj.r2121 41093619

[6] Morel A, Meuleman M-S, Moktefi A et al. Renal diseases associated with hematologic malignancies and thymoma in the absence of renal monoclonal immunoglobulin deposits. Diagnostics. 2021;11:710. 10.3390/diagnostics11040710 33921123 PMC8071536

[7] Marques O, Weiss G, Muckenthaler MU. The role of iron in chronic inflammatory diseases: from mechanisms to treatment options in anemia of inflammation. Blood. 2022;140:2011–23. 10.1182/blood.2021013472 35994752

[8] Lanas A, Andrews JM, Lau J et al. Management of iron-deficiency anemia following acute gastrointestinal hemorrhage: a narrative analysis and review. J Gastro Hepatol. 2023;38:23–33. 10.1111/jgh.16033 36266733

[9] Cases A, Cigarrán S, Luis Górriz J et al. Effect of SGLT2 inhibitors on anemia and their possible clinical implications. Nefrología (English Edition). 2024;44:165–72. 10.1016/j.nefroe.2024.03.011 38604895

[10] Ekanayake P, Mudaliar S. Increase in hematocrit with SGLT-2 inhibitors—hemoconcentration from diuresis or increased erythropoiesis after amelioration of hypoxia?. Diabetes Metab Syndr. 2023;17:102702. 10.1016/j.dsx.2022.102702 36657305

[11] Caulier AL, Sankaran VG. Molecular and cellular mechanisms that regulate human erythropoiesis. Blood. 2022;139:2450–9. 10.1182/blood.2021011044 34936695 PMC9029096

[12] Angermann CE, Santos-Gallego CG, Requena-Ibanez JA et al. Empagliflozin effects on iron metabolism as a possible mechanism for improved clinical outcomes in non-diabetic patients with systolic heart failure. Nat Cardiovasc Res. 2023;2:1032–43. 10.1038/s44161-023-00352-5 39196095 PMC11358002

[13] Nakai T, Iwamura Y, Kato K et al. Drugs activating hypoxia-inducible factors correct erythropoiesis and hepcidin levels via renal EPO induction in mice. Blood Adv. 2023;:7;:3793–805. 10.1182/bloodadvances.2023009798 37146271 PMC10393763

[14] Gao Y-M, Feng S-T, Wen Y et al. Cardiorenal protection of SGLT2 inhibitors—perspectives from metabolic reprogramming. eBioMedicine. 2022;83:104215. 10.1016/j.ebiom.2022.104215 35973390 PMC9396537

[15] Jin Z, Yin R, Yuan Y et al. Dapagliflozin ameliorates hepatic steatosis via suppressing LXRα-mediated synthesis of lipids and bile acids. Biochem Pharmacol. 2024;223:116167. 10.1016/j.bcp.2024.116167 38527558

[16] Behera N, Bhattacharyya G, Behera S et al. Iron mobilization from intact ferritin: effect of differential redox activity of quinone derivatives with NADH/O(2) and *in situ*-generated ROS. J Biol Inorg Chem. 2024;29:455–75. 10.1007/s00775-024-02058-w 38780762

[17] Perkovic V, Jardine MJ, Neal B et al. Canagliflozin and renal outcomes in type 2 diabetes and nephropathy. N Engl J Med. 2019;380:2295–306. 10.1056/NEJMoa1811744 30990260

[18] Waijer SW, Vart P, Cherney DZI et al. Effect of dapagliflozin on kidney and cardiovascular outcomes by baseline KDIGO risk categories: a post hoc analysis of the DAPA-CKD trial. Diabetologia. 2022;65:1085–97. 10.1007/s00125-022-05694-6 35445820 PMC9174107

[19] Empagliflozin in patients with chronic kidney disease. N Engl J Med. 2023;388:117–27. 10.1056/NEJMoa2204233 36331190 PMC7614055

[20] Heerspink HJL, Stefánsson BV, Correa-Rotter R et al. Dapagliflozin in patients with chronic kidney disease. N Engl J Med. 2020;383:1436–46. 10.1056/NEJMoa2024816 32970396

[21] Morales E, Galindo M. SGLT2 inhibitors in lupus nephropathy, a new therapeutic strategy for nephroprotection. Ann Rheum Dis. 2022;81:1337–8. 10.1136/annrheumdis-2022-222512 35551062

[22] Zhao X-Y, Li S-S, He Y-X et al. SGLT2 inhibitors alleviated podocyte damage in lupus nephritis by decreasing inflammation and enhancing autophagy. Ann Rheum Dis. 2023;82:1328–40. 10.1136/ard-2023-224242 37487609

[23] Bertsias GK, Tektonidou M, Amoura Z et al. Joint European League Against Rheumatism and European Renal Association-European Dialysis and Transplant Association (EULAR/ERA-EDTA) recommendations for the management of adult and paediatric lupus nephritis. Ann Rheum Dis. 2012;71:1771–82. 10.1136/annrheumdis-2012-201940 22851469 PMC3465859

[24] Hanly JG, O’Keeffe AG, Su L et al. The frequency and outcome of lupus nephritis: results from an international inception cohort study. Rheumatology. 2016;55(2):252–62. 10.1093/rheumatology/kev311 26342222 PMC4939728

[25] Parikh SV, Almaani S, Brodsky S et al. Update on lupus nephritis: core curriculum 2020. Am J Kidney Dis. 2020;76:265–81. 10.1053/j.ajkd.2019.10.017 32220510

[26] Sinha SK, Nicholas SB. Pathomechanisms of diabetic kidney disease. J Clin Med. 2023;12:7349. 10.3390/jcm12237349 38068400 PMC10707303

[27] Cherney DZI, Dekkers CCJ, Barbour SJ et al. Effects of the SGLT2 inhibitor dapagliflozin on proteinuria in non-diabetic patients with chronic kidney disease (DIAMOND): a randomised, double-blind, crossover trial. Lancet Diabetes Endocrinol. 2020;8:582–93. 10.1016/S2213-8587(20)30162-5 32559474

[28] Heerspink HJL, Kosiborod M, Inzucchi SE et al. Renoprotective effects of sodium-glucose cotransporter-2 inhibitors. Kidney Int. 2018;94:26–39. 10.1016/j.kint.2017.12.027 29735306

[29] van Bommel EJM, Muskiet MHA, van Baar MJB et al. The renal hemodynamic effects of the SGLT2 inhibitor dapagliflozin are caused by post-glomerular vasodilatation rather than pre-glomerular vasoconstriction in metformin-treated patients with type 2 diabetes in the randomized, double-blind RED trial. Kidney Int. 2020;97:202–12. 31791665 10.1016/j.kint.2019.09.013

[30] Herrington WG, Staplin N, Wanner C et al. Empagliflozin in patients with chronic kidney disease. N Engl J Med. 2023;388:117–27. 36331190 10.1056/NEJMoa2204233 PMC7614055

[31] Yau K, Dharia A, Alrowiyti I et al. Prescribing SGLT2 inhibitors in patients with CKD: expanding indications and practical considerations. Kidney Int Rep. 2022;7:1463–76. 10.1016/j.ekir.2022.04.094 35812300 PMC9263228

[32] Qiao P, Jia Y, Ma A et al. Dapagliflozin protects against nonalcoholic steatohepatitis in db/db mice. Front Pharmacol. 2022;13:934136. 10.3389/fphar.2022.934136 36059948 PMC9437261

[33] Kanbay M, Tapoi L, Ureche C et al. Effect of sodium-glucose cotransporter 2 inhibitors on hemoglobin and hematocrit levels in type 2 diabetes: a systematic review and meta-analysis. Int Urol Nephrol. 2022;54:827–41. 10.1007/s11255-021-02943-2 34273060

[34] Docherty KF, Welsh P, Verma S et al. Iron deficiency in heart failure and effect of dapagliflozin: findings from DAPA-HF. Circulation. 2022;146:980–94. 10.1161/CIRCULATIONAHA.122.060511 35971840 PMC9508991

[35] Oshima M, Neuen BL, Jardine MJ et al. Effects of canagliflozin on anaemia in patients with type 2 diabetes and chronic kidney disease: a post-hoc analysis from the CREDENCE trial. Lancet Diabetes Endocrinol. 2020;8:903–14. 10.1016/S2213-8587(20)30300-4 33065060

[36] Yen FS, Wang SI, Hsu CC et al. Sodium-glucose cotransporter-2 inhibitors and nephritis among patients with systemic lupus erythematosus. JAMA Netw Open. 2024;7:e2416578. 10.1001/jamanetworkopen.2024.16578 38865122 PMC11170305

[37] Mazer CD, Hare GMT, Connelly PW et al. Effect of empagliflozin on erythropoietin levels, iron stores, and red blood cell morphology in patients with type 2 diabetes mellitus and coronary artery disease. Circulation. 2020;141:704–7. 10.1161/CIRCULATIONAHA.119.044235 31707794

[38] Packer M . Mechanisms of enhanced renal and hepatic erythropoietin synthesis by sodium-glucose cotransporter 2 inhibitors. Eur Heart J. 2023;44:5027–35. 10.1093/eurheartj/ehad235 37086098 PMC10733737

[39] Ghanim H, Abuaysheh S, Hejna J et al. Dapagliflozin suppresses hepcidin and increases erythropoiesis. J Clin Endocrinol Metab. 2020;105:e1056–63. 10.1210/clinem/dgaa057 32044999

[40] Ueda N, Takasawa K. Impact of inflammation on ferritin, hepcidin and the management of iron deficiency anemia in chronic kidney disease. Nutrients. 2018;10:1173. 10.3390/nu10091173 30150549 PMC6163440

